# Derived cannabinoid product availability among online vape shops

**DOI:** 10.1016/j.pmedr.2024.102910

**Published:** 2024-10-22

**Authors:** Nora Satybaldiyeva, Raquel Harati, Tomas Mejorado, Nicolas Morales, Gustavo Benitez, Shannon E. Ellis, Karen Ferran, Eric C. Leas

**Affiliations:** a Herbert Wertheim School of Public Health and Human Longevity Science, University of California San Diego, La Jolla, CA, USA; b School of Public Health, San Diego State University, San Diego, CA, USA; c California State University San Marcos, San Marcos, CA, USA; d Department of Cognitive Science, University of California San Diego, La Jolla, CA, USA; e Halıcıoğlu Data Science Institute, University of California San Diego, La Jolla, CA, USA; f Qualcomm Institute, University of California San Diego, La Jolla, CA, USA

**Keywords:** Cannabinoids, Vape shops, Policy, Retail, Compliance

## Abstract

**Objectives:**

To determine the proportion of online vape shops that offer derived cannabinoid products in a large metropolitan area (San Diego, California), measure their compliance with state hemp regulations, and estimate whether these shops receive more website traffic compared to those that offered nicotine or tobacco.

**Methods:**

We obtained vape shops (n = 109) using browser-based (i.e., Google Search) and map-based (i.e., Google Maps and Yelp) searches in San Diego from March to August 2023 and conducted a content analysis of their websites to identify derived cannabinoid products offered for mail-order purchasing. Using website traffic data, we examined the association between derived cannabinoid product availability and monthly website visits.

**Results:**

Among the 109 online vape shops in the study sample, 35.8 % offered derived cannabinoid products for mail-order purchase and 26.6 % sold flavored (excluding terpenes) and inhalable cannabinoid products. Compared to vape shops that did not offer derived cannabinoid products (Mean: 23,619; 95 % CI: 23,605–23,634), those offering flavored and inhalable cannabinoid products received 2.5 times more monthly website visits (Mean: 57,950; 95 % CI: 57,913–57,986) and those offering any derived cannabinoid products received 5.5 times more monthly website visits (Mean: 130,694; 95 % CI: 130,607–130,782).

**Conclusion:**

The prevalence of flavored and inhalable derived cannabinoid products, despite being prohibited under California’s hemp regulations, highlights the need for additional enforcement measures to ensure retailer compliance. The higher website traffic observed among vape shops offering cannabinoid products may be indicative of greater popularity of retailers selling derived cannabinoid products compared to those selling nicotine or tobacco products alone.

## Highlights

• Nearly 36% of vape shops in San Diego, California sold derived cannabinoid products.
• About 27% of vape shops sold flavored and inhalable derived cannabinoid products.
• California’s Assembly Bill 45 prohibits flavored & inhalable derived cannabinoid sale.
• Vape shops that sold derived cannabinoid products received more website visits.

## 1 Introduction

Cannabinoids are chemical compounds that naturally occur at various levels in all species of *Cannabis Sativa L.* (Hanuš et al., 2016). However, since the passage of the 2018 Farm Bill in the United States (US), *Cannabis Sativa L.* and its derived cannabinoids can be legally classified as either “hemp” or “marijuana” based on their concentration of delta-9-tetrahydrocannabinol (delta-9-THC), with plants or derivatives containing ≤ 0.3 % delta-9-THC considered “hemp” and those with > 0.3 % delta-9-THC considered “marijuana” (i.e., cannabis) (Agriculture and T.U.S.S.C.O. , 2018). A critical drug policy question is whether it is legal to sell cannabinoids derived from hemp to produce psychotropic chemical analogs to delta-9-THC, such as $\Delta^{8}$ tetrahydrocannabinol (delta-8-THC) (Dickson et al., 2019). Many manufacturers have interpreted the 2018 Farm Bill as implying that hemp and marijuana derivatives are legal so long as they contain ≤ 0.3 % delta-9-THC. However, the Drug Enforcement Agency has refuted this assumption in some communications but has yet to take broad-sweeping actions against hemp and marijuana derived product manufacturers (Leas, 2021). This legal ambiguity about products like delta-8-THC has created a new market for ‘derived’ cannabinoid products in the US (Kruger and Kruger, 2021, Kruger and Kruger, 2022, Leas et al., 2022, Livingston et al., 2022, Rossheim et al., 2023, Rossheim et al., 2022).

The availability of derived cannabinoid products in inhalable modalities, such as vapes, has created regulatory complexities that overlap with tobacco regulation. Almost all retailers in Fort Worth, Texas with an alcohol, tobacco, or cannabidiol (CBD) license that sold delta-8-THC (96 %) offered product types that were intended to be smoked or vaped (Rossheim et al., 2022). Furthermore, an assessment of online retailers of psychotropic cannabinoid products showed that most of the modalities offered were inhalable, such as disposable vapes (43 %), vape carts (18 %), and pre-rolls (7 %) (Rossheim et al., 2024b). The overlap in modes of use between tobacco and cannabinoid products can interfere with efforts to pinpoint culprit products during vaping-related outbreaks, such as the e-cigarette or vaping-associated lung injuries outbreak, in which many cases vaped a combination of cannabinoid and tobacco products (Blount et al., 2020, Heinzerling et al., 2020). Although tobacco product manufacturers are required to submit product applications to the Food and Drug Administration (FDA) before they can market and distribute them in the US, the FDA does not have a similar framework for derived cannabinoid products. Nevertheless, the FDA has taken some actions to protect public health by issuing warning letters to companies marketing inhalable CBD products and delta-8-THC products (Wagoner et al., 2021). The interchangeability of vaping products can also create loopholes in regulation. For example, although several US states have banned flavored nicotine vapes to tackle nicotine vaping among youth, similar regulations for derived cannabinoid products are rare (Egan et al., 2023), even though cannabinoid product use is prevalent among US youth (Harlow et al., 2024).

The lack of quality and safety standards for derived cannabinoid products may also have public health impacts. Without established quality assurance standards, derived cannabinoid products can be mislabeled, may include more than 0.3 % delta-9-THC, and may include harmful contaminants (Gidal et al., 2024, Meehan-Atrash and Rahman, 2021). For example, one assessment of commercially available CBD products in the US found that most (74 %) of these products were inaccurately labeled, and several contained heavy metals, residual solvents, and pesticides that violated regulatory thresholds (Gidal et al., 2024). Similarly, a toxicological analysis of 27 vaping products containing delta-8-THC found that all of the products had mislabeled the delta-8-THC content contained and contained side-products such as heavy metals, and many products contained unlabeled cutting agents (Meehan-Atrash and Rahman, 2021). There were over 2,000 reports of adverse events linked to delta-8-THC consumption received by the FDA and the National Poison Control Centers between December 2020 and February 2022, many of which required medical intervention and involved pediatric patients (Administration, 2022, Simon et al., 2023, Staff, 2021). Many of the symptoms presented in adverse events related to delta-8-THC use were similar to those reported for acute delta-9-THC intoxication (e.g., anxiety and paranoia), but there were several unique symptoms that could have stemmed from unregulated manufacturing methods (e.g., muscle spasms) (Leas et al., 2023a).

Given the existing regulatory challenges and health risks associated with derived cannabinoid products, very little is known about how vape shops, specifically those with an online presence, have responded to their growing popularity. Prior research has already shown that tobacco retailers, including vape shops, often sell substances other than nicotine, including kratom, CBD, and delta-8-THC (Bowdring et al., 2023, Kruger and Kruger, 2021, Leas et al., 2021, LoParco et al., 2023, Rossheim et al., 2022). A recent review of 520 vape shops across the US found that 74 % offered psychotropic cannabinoid products for sale (Rossheim et al., 2024). Despite local regulations for flavored tobacco products (Leas et al., 2023b), online vape shops may be introducing inhalable cannabinoid products with characterizing flavors as a new substance that is appealing to youth, even though they may pose increased short-term risks. Online retailers, which frequently use e-commerce platforms, are often not included in the definition of “retailers” within local and state policies governing tobacco products (Leas, 2024). Therefore, online retailers may operate across state lines, potentially avoiding local regulations, such as state bans on flavored tobacco products. There are jurisdictions in which online retailers are subject to laws governing certain products. For example, California’s Assembly Bill 45 prohibits the manufacture or sale of inhalable industrial hemp products (i.e., cannabis plants containing ≤ 0.3 % delta-9-THC), including retailers that offer products through a delivery service (i.e., e-commerce), except for the sole purpose of sale in other states. As a part of this law, inhalable industrial hemp products, including those sold across state lines, cannot contain characterizing flavors other than natural terpenes, may not be sold to consumers under 21 years of age, and may not be sold in retail locations in California (AB45, C., 2021). However, to our knowledge, no surveillance of flavored and inhalable cannabinoid products to inform enforcement activity has been conducted.

To inform policy around cannabinoid products, we describe the availability of derived cannabinoid products in online tobacco retailers in California. Furthermore, we evaluate compliance with a local law that restricts flavored and inhalable hemp product sales. Since vape shops may be offering derived cannabinoid products as a way of generating business, we used web traffic data for the websites in our study sample to compare traffic according to whether they offer derived cannabinoid products or not.

## 2 Methods

### 2.1 Study sample

**Fig. 1:**
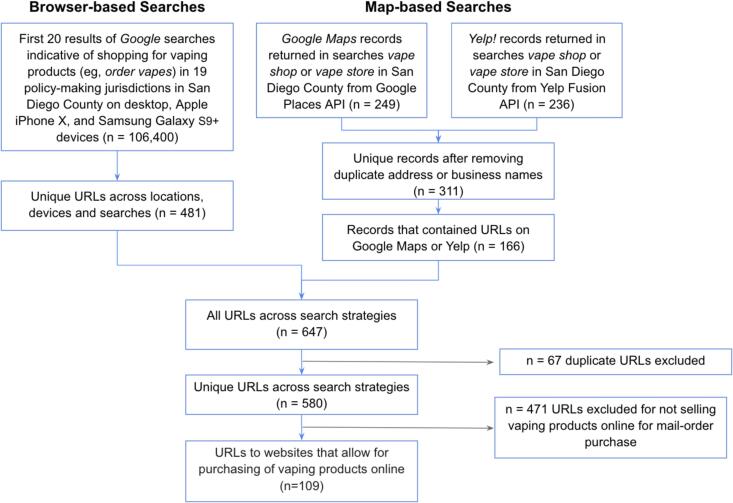
Derivation of study sample of online vape shops offering products for mail-order purchase serving San Diego, California, from March to August 2023. Abbreviations: URLs, Uniform Resource Locators; API, Application Programming Interfaces.

We obtained a comprehensive sample of vape shops with e-commerce websites in San Diego County, California from March to August of 2023 (Fig. 1). The comprehensive sample included retailers that had a website and a physical retail location in San Diego County (“map-based”) as well as retailers with a website returned in a search conducted in San Diego County (“browser-based”).

Map-based retailers were identified using the Google Maps and Yelp Application Programming Interfaces with locations set in San Diego County. We used a predefined store type category called “vape shops” to retrieve retailers from Yelp. For Google Maps, we used the following search terms as inputs: “ecig”, “e-cig”, “vape”, “vapor”, “vaper”, “vapin,” “vaporizer store”, “vape shop”, and “vape store.” Two separate lists of vape shops from Yelp and Google Maps were merged using street addresses and zip codes to link common records. The merged dataset consisted of 311 unique vape shops, of which 166 contained uniform resource locators (URLs) to a website.

Browser-based retailers were collected using Google searches for 5 terms indicative of shopping, including “buy”, “order”, “shop”, “retailer”, or “sale”, in combination with a vaping or product-indicative term (i.e., “vape”, “e-cig”, “JUUL”, “NJOY”). Searches were performed using a Google Chrome browser with the location of searches positioned to the geographic center point of each jurisdiction within San Diego County. Google searches returned in the first two pages of results for each search term combination initially yielded 106,400 search results. After simplifying results to only include top-level URLs (i.e. the top-level URL for https://www.sciencedirect.com/journal/preventive-medicine-reports is sciencedirect.com) and removing duplicates, 481 were unique URLs.

After merging the unique URLs across search strategies (n = 580) and removing URLs to websites that did not allow for mail-order purchasing of vape products (n = 471), we retained 109 online vape shops for further content analysis. Of the 109 online vape shops in our study sample, 26 were obtained from map-based searches and 83 were obtained from browser-based searches.

### 2.2 Content analysis

We visited all the e-commerce websites included in our final analytic sample to conduct a content analysis. Following a previous protocol used to study online vaping retailers and other online content discussing delta-8-THC (e.g., Reddit posts), we developed a codebook and four independent, trained coders (N.S., R.H., T.M., and N.M) working in teams of two coded websites to determine whether and what type of cannabinoid products the retailer sold while using an automated archiving software (Hunchly, 2024). The automated archiving software allowed coders to capture screenshots of web pages (including text, images, and web page structure) for each website and then tag all the relevant content to the retailer identifier. After review, we calculated agreement between the two coders, (Viera and Garrett, 2005) flagged disagreements by comparing labels, and reviewed and adjudicated (R.H.) any websites with discordance using the saved archive.

### 2.3 Derived cannabinoid product availability

Using the list of all retailers (map-based and browser-based), we identified retailers with e-commerce websites that allowed for mail-order purchasing of derived cannabinoid products. We coded the following information: “Does the website sell hemp-derived products?” with response options “yes” and “no”. If a website was coded as selling cannabinoid products, 10 additional questions were asked to collect information about the different types of cannabinoid products available. Each of the 10 questions was presented in a similar format, “Does the website sell… 1) Cannabidiol (CBD), 2) Δ8-Tetrahydrocannabinol (delta-8-THC), 3) Hexahydrocannabinol (HHC), 4) Tetrahydrocannabinol acetate (THC-O), 5) Δ10 Tetrahydrocannabinol (delta-10-THC), 6) Cannabinol (CBN), 7) Cannabigerol (CBG), 8) Hemp products (cannabinoids not defined), 9) Hemp legal Δ9-Tetrahydrocannabinol (Containing < 0.3 % delta-9-THC), and 10) Other Product not mentioned.” General derived products that did not specify which cannabinoids they contained (e.g., full-spectrum hemp) were coded as “yes” for “8) Hemp products (cannabinoids not defined)” while derived products that contained a cannabinoid not listed (e.g., Tetrahydrocannabiphorol) were coded as “yes” for “10) Other Product not mentioned”. If a product contained a blend of multiple cannabinoids, each cannabinoid listed in the blend was coded separately. There was high agreement between the two pairs of independent coders for all the cannabinoid product-related measures (Supplemental Table S1). Coder agreement for the availability of specific cannabinoids in vape shops was high, with prevalence-bias-adjusted kappas ranging from 0.80 to 1.00.

### 2.4 Inhalable and flavored derived cannabinoid products

Among websites selling derived cannabinoid products, we collected information on whether any of the cannabinoid products offered by the retailer were inhalable (i.e., vapes, e-liquids, cartridges, flower, pre-rolls, and concentrates) and contained a characterizing flavor (e.g., “honeydew ice”). Cannabinoid products containing natural terpene flavors only (e.g., limonene) were not coded as flavored because they are permitted under California’s Assembly Bill 45. Products described with ‘non-flavor’ flavors (e.g., Northern Lights) were also not coded as flavored because they do not reflect conventional taste expectations (LoParco et al., 2024). Flavored and inhalable cannabinoid product availability was assessed similarly to cannabinoid product availability using 10 separate questions for each of the derived cannabinoid products listed above. To avoid burdening annotators and assess compliance with Assembly Bill 45, each of the 10 questions simultaneously asked whether a product was inhalable and flavored. There was high coder agreement for inhalable and flavored varieties of each cannabinoid, with prevalence-bias-adjusted kappas ranging from 0.76 to 0.93.

### 2.5 Website traffic

We purchased a six-month report from Semrush (https://www.semrush.com) that provided an estimate of the number of monthly website visits to each website in our final analytic sample (March 2023 through August 2023). Semrush calculated the estimated monthly visits nationally for each website. Across the six months reflected in the report, there was a total of 28,193,827 website visits directed from Google searches to the websites in our sample.

### 2.6 Statistical analyses

We calculated the percentage of retailers that offered derived cannabinoid products for mail-order purchasing. Among retailers that permitted mail-order purchasing of cannabinoid products, we identified the proportion of vape shops that offered inhalable cannabinoid products with non-terpene-derived flavors and were, therefore, non-compliant with California’s Assembly Bill 45.

To compare the website traffic of retailers that offered cannabinoid products and those that did not, we ran a hierarchical Poisson regression model with web traffic as the dependent variable, availability of cannabinoid products as the independent variable, and adjustment for retailer type. Web traffic was operationalized as a continuous variable measured for each month over the six-month period for each retailer and the retailer ID variable was included to account for the fact that the number of visits were grouped by retailer. Cannabinoid product availability was a three-level variable categorized as 1) none, 2) inhalable and flavored products offered, or 3) cannabinoid products offered but not inhalable and flavored. Retailer type was a dichotomous variable categorized as map-based or browser-based. We also report the web traffic estimated marginal means and corresponding 95 % confidence intervals for the three-level cannabinoid product availability variable by retailer type. As a part of our sensitivity analyses, we assessed the association between offering any derived cannabinoid products and monthly website visits, and among retailers that offered derived cannabinoid products, whether offering inhalable and flavored modalities was associated with website visits. The University of California, San Diego Institutional Review Board did not consider this study human subjects research. All analyses were performed using R V.3.6.1 and ɑ=0.05.

## 3 Results

Among the 109 online vape shops in the study sample, 35.8 % offered a derived cannabinoid product for mail-order purchase (Table 1). The most common cannabinoids listed as main ingredients among the retailers were CBD (32.1 %), delta-8-THC (20.2 %), hemp-derived delta-9-THC (17.4 %), HHC (16.5 %), CBG (14.7 %), delta-10-THC (13.8 %), THC-O (10.1 %), and CBN (10.1 %). There were 12 “other” cannabinoid products sold by the vape shops including Tetrahydrocannabiphorol (10.1 %), Hexahydrocannabiphorol (3.7 %), Delta-9 tetrahydrocannabutol (3.7 %), Tetrahydrocannabinolic acid (2.8 %), Delta-6-THC (2.8 %), THC-X (1.8 %), Delta-11-THC (2.8 %), Cannabidivarin (0.9 %), Delta-7-THC (0.9 %), Tetrahydrocannabinol monomethylether (0.9 %), Hydrox-4-phc (0.9 %), and Tetrahydrocannabivarin (0.9 %).

**Table 1:** Distribution of cannabinoids listed as main ingredients among all online vape shops serving San Diego, California, from March to August 2023, by product type (n = 109).

**Cannabinoid**	**Any products** *column %*	**Flavored and inhalable products** *column %*
Any	35.8	26.6
Cannabidiol (CBD)	32.1	21.1
Delta-8- tetrahydrocannabinol (THC)	20.2	18.3
Hemp-derived delta-9-THC	17.4	13.8
Hexahydrocannabinol (HHC)	16.5	14.7
Cannabigerol (CBG)	14.7	7.3
Delta-10-THC	13.8	13.8
Tetrahydrocannabinol acetate (THCO)	10.1	11.0
Cannabinol (CBN)	10.1	9.2
Tetrahydrocannabiphorol (THCP)	10.1	10.1
Hexahydrocannabiphorol (HHCP)	3.7	3.7
Delta-9 tetrahydrocannabutol (THCB)	3.7	3.7
Tetrahydrocannabinolic acid (THCA)	2.8	2.8
Delta-6-THC	2.8	2.8
THC-X	1.8	1.8
Delta-11-THC	2.8	2.8
Cannabidivarin (CBDV)	0.9	0.9
Delta-7-THC	0.9	0.9
Tetrahydrocannabinol monomethylether (THCM)	0.9	0.9
Hydrox-4-phc (PHC)	0.9	0.9
Tetrahydrocannabivarin (THCV)	0.9	0.9

Note: THC-X is a manufactured cannabinoid.

Over a quarter of the retailers (26.6 %) sold flavored and inhalable derived cannabinoid products (Table 1). Each of the cannabinoids identified was offered in flavored and inhalable form within our sample of online vape shops. There were 21.1 % of retailers offering flavored and inhalable products containing CBD, 18.3 % containing delta-8-THC, 14.7 % containing HHC, 13.8 % containing hemp-derived delta-9-THC and delta-10-THC, 11.0 % containing THC-O, 9.2 % containing CBN, and 7.3 % containing CBG. Nearly three-fourths (74.4 %) of online vape shops that offered derived cannabinoid products offered them in flavored and inhalable form.

Online vape shops that sold inhalable and flavored derived cannabinoid products received 2.5 times more average monthly website visits (Mean: 57,950; 95 % CI: 57,913–57,986) than vape shops that did not offer any cannabinoid products for sale (Mean: 23,619; 95 % CI: 3,605–23,634). Similarly, vape shops that sold cannabinoid products, not including those that were inhalable and flavored, had average monthly visit rates that were 5.5 times higher (Mean: 130,694; 95 % CI: 130,607–130,782) than vape shops that did not sell any cannabinoid products. The association between cannabinoid product availability and average website visits was similar across the two types of retailers examined within the study sample (Table 2). After adjusting for retailer type, offering cannabinoid products, both inhalable and flavored and not inhalable and flavored, was positively associated with a greater number of monthly website visits compared to not offering any cannabinoid products (Supplemental Table 2). Our sensitivity analyses showed that offering any derived cannabinoid products was positively associated with a greater number of monthly website visits compared to not offering any derived cannabinoid products (Supplemental Table 3). However, among retailers that offered derived cannabinoid products, offering inhalable and flavored modalities was not associated with monthly website visits (Supplemental Table 4).

**Table 2:** Average monthly website traffic for categories of cannabinoid product availability, overall and by retailer type, among online vape shops serving San Diego, California, from March to August 2023 (n = 109).

	**Overall**	**Map-Based Retailers**	**Browser-Based** **Retailers**
**Cannabinoid Products Offered**	EMM (95 % CI)	EMM (95 % CI)	EMM (95 % CI)
None	23,619 (23,605–23,634)	1,306 (1,302–1,310)	30,914 (30,895–30,934)
Inhalable and Flavored	57,950 (57,913–57,986)	2,953 (2,943–2,962)	69,906 (69,862–69,950)
Not Inhalable or Flavored	130,694 (130,607–130,782)	8,470 (8,444–8,496)	200,537 (200,403–200,672)

Abbreviations: EMM, Estimated Marginal Mean; CI, Confidence interval.

## 4 Discussion

We found that just over a third of online vape shops serving San Diego County, California offered derived cannabinoid products and just over a quarter offered flavored and inhalable derived cannabinoid products, despite these being prohibited under California’s Assembly Bill 45. The majority of online vape shops that offered derived cannabinoid products were out of compliance with California’s Assembly Bill 45. Vape shops that offered derived cannabinoid products received significantly more monthly website visits compared to those that did not offer any cannabinoid products, suggesting that selling cannabinoid products may be associated with website popularity and there is substantial consumer interest in these products.

The availability of derived cannabinoid products among our sample of online vape shops in San Diego County is consistent with previous research on CBD and delta-8-THC products in retail settings (Leas et al., 2021, Rossheim et al., 2024, Rossheim et al., 2022). For example, a study from 2021 found that 37 % of online vape shops sold products containing CBD, with the majority offering inhalable products, which is similar to the proportion of retailers that offered a cannabinoid product for mail-order purchase in our sample (Leas et al., 2021). Among a sample of map-based retailers in Fort Worth, Texas in 2021, 11 % reported selling delta-8-THC, a much smaller proportion than that found within our sample (20.2 %) (Rossheim et al., 2022). This discrepancy may be due to their inclusion of retail locations with alcohol and CBD licenses, resulting in a larger sample of total retailers. Lastly, our results show that derived cannabinoid product availability among online vape shops is similar to those with a physical location, as a recent study of vape shops in the US found that 50 % of vape shops with a physical location in California sold intoxicating cannabinoid products (Rossheim et al., 2024).

More than one in four online vape shops in San Diego were non-compliant with California’s Assembly Bill 45, which pertains to industrial hemp products, as they offered derived cannabinoid products that were inhalable and flavored. One mechanism for addressing non-compliance may be through an already established model of compliance monitoring used by the County of San Diego Tobacco Retail Licensing program. Furthermore, online vape shops out of compliance with Assembly Bill 45 had greater website traffic than those not offering any cannabinoid products for sale, which may be indicative of their increased popularity.

Our finding of a dozen “other” derived cannabinoid products sold by online vape shops raises several concerns given that there is scarce scientific literature on these products and their safety profile. Given that previous research has shown several adverse events associated with delta-8-THC use, the use of these “other” cannabinoid products, many of which are mildly psychoactive, may result in similar negative health outcomes, such as anxiety, paranoia, and respiratory issues (Leas et al., 2023a). Similar to popular cannabinoids, such as delta-8-THC, there is likely a lack of regulatory oversight and quality control in the production of these “other” cannabinoid products.

There are several strengths and limitations to the current study. A notable strength is our use of a comprehensive sample that includes retailers with websites returned in the first two pages of a Google Search in San Diego County, as previous research has shown that over 70 % of users click a link on the first page of Google results (Petrescu et al., 2015). However, this approach may introduce sampling bias, as more frequently visited websites may be more likely to have been included in our study sample. Additionally, the restriction of our sample to San Diego County limits the generalizability of our findings. Given that psychotropic derived cannabinoid products are widely available in vape shops throughout the US (Rossheim et al., 2024), our study findings need to be replicated in other states with different regulations around the sale of derived cannabinoid products. The use of vape-related terms for our browser-based and map-based searches may have also biased our study sample to include a greater proportion of inhalable derived cannabinoid products. An additional strength of the current study is the ability to measure the association between website visits and the types of products available for sale by that website. Although the measure of website traffic allows us to expand on previous research in the field of e-commerce tobacco retailer surveillance, the 6-month measure of website visits in the current study serves as a proxy for vape shop popularity. We did not measure the number of sales or purchases made for any of the websites in the current study.

### 4.1 Conclusion

We found that a sizeable proportion of online vape shops sold derived cannabinoid products, most of which were psychotropic. Most online vape shops that sold cannabinoid products offered them in flavored and inhalable modalities, which are prohibited under California’s Assembly Bill 45, which governs industrial hemp products. Notably, vape shops that offered cannabinoid products had a higher number of monthly website visits, which may underscore their popularity as well as their ability to attract a broader range of consumers, including those interested in cannabis products. The availability of cannabinoid products among online vape shops raises multiple public health and policy concerns, such as the lack of standardized safety measures (i.e., limits on total THC content) and the absence of marketing regulations for these products. Given that the health effects of many derived cannabinoid products are unknown, and their use is associated with poisonings and other adverse events, additional research is needed to establish their risk profile. Our findings highlight the need for strengthened regulations of online vape shops offering cannabinoid products to prevent potential public health issues resulting from their sale.

## CRediT authorship contribution statement

**Nora Satybaldiyeva:** Writing – review & editing, Writing – original draft, Formal analysis, Data curation. **Raquel Harati:** Writing – review & editing, Project administration, Data curation. **Tomas Mejorado:** Writing – review & editing, Data curation. **Nicolas Morales:** Writing – review & editing, Data curation. **Gustavo Benitez:** Writing – review & editing. **Shannon E. Ellis:** Writing – review & editing, Methodology. **Karen Ferran:** Writing – review & editing. **Eric C. Leas:** Writing – review & editing, Writing – original draft, Project administration, Investigation, Funding acquisition, Conceptualization.

## Declaration of competing interest

The authors declare that they have no known competing financial interests or personal relationships that could have appeared to influence the work reported in this paper.

## Data Availability

The data underlying this article will be shared on reasonable request to the corresponding author.
