# Supplementary material for: Derived cannabinoid product availability among online vape shops
Source: Prev Med Rep. 2024 Oct 22;48:102910. doi: 10.1016/j.pmedr.2024.102910 (PMC11541839; doi:10.1016/j.pmedr.2024.102910)
Supplement: Supplementary Data 1 [file mmc1.docx]

**Supplemental Tables**

**Supplemental Table 1:** Agreement and reliability between two independent coders for cannabinoid product-related measures assessed among online vape shops serving San Diego, California, from March to August 2023.

| **Cannabinoid** | **Agreement**  **(***%)* | **adjusted kappa**† |
| --- | --- | --- |
| Any | 0.91 | 0.82 |
| Cannabidiol (CBD) | 0.90 | 0.80 |
| *Inhalable + Flavored CBD* | 0.94 | 0.89 |
| Delta-8- tetrahydrocannabinol (THC) | 0.96 | 0.93 |
| *Inhalable + Flavored delta-8-THC* | 0.95 | 0.91 |
| Hexahydrocannabinol (HHC) | 0.96 | 0.93 |
| *Inhalable + Flavored HHC* | 0.95 | 0.91 |
| Tetrahydrocannabinol acetate (THCO) | 0.96 | 0.93 |
| *Inhalable + Flavored THCO* | 0.96 | 0.93 |
| Delta-10-THC | 0.95 | 0.91 |
| *Inhalable + Flavored delta-10-THC* | 0.94 | 0.89 |
| Cannabinol (CBN) | 0.95 | 0.91 |
| *Inhalable + Flavored CBN* | 0.95 | 0.91 |
| Cannabigerol (CBG) | 0.95 | 0.91 |
| *Inhalable + Flavored CBG* | 0.94 | 0.89 |
| Hemp-compliant delta-9-THC | 0.93 | 0.85 |
| *Inhalable + Flavored hemp-compliant delta-9-THC* | 0.93 | 0.85 |
| Other | 1.00 | 1.00 |
| *Inhalable + Flavored other cannabinoid* | 0.88 | 0.76 |

†The kappa statistic is adjusted for the low prevalence of cannabinoid product availability among vape shops in the study sample.

**Supplemental Table 2:** Association between types of cannabinoid products offered and average monthly website traffic, adjusting for retailer type among online vape shops serving San Diego, California, from March to August 2023 (n=109).

|  | Beta | Standard error | | t value | | p-value |
| --- | --- | --- | --- | --- | --- | --- |
| **Cannabinoid Products Offered** |  |  |  | |  | |
| None | Ref |  | |  | |  |
| Inhalable and Flavored | 30348 | 11932 | | 2.54 | | 0.011 |
| Not Inhalable or Flavored | 113962 | 17306 | | 6.59 | | <0.001 |
| **Retailer Type** |  |  | |  | |  |
| Map-based | Ref |  | |  | |  |
| Browser-based | 58734 | 12042 | | 4.88 | | <0.001 |

**Supplemental Table 3:** Association between derived cannabinoid product availability and average monthly website traffic, adjusting for retailer type among online vape shops serving San Diego, California, from March to August 2023 (n=109).

|  | Beta | Standard error | | t value | | p-value |
| --- | --- | --- | --- | --- | --- | --- |
| **Cannabinoid Products Offered** |  |  |  | |  | |
| No | Ref |  | |  | |  |
| Yes | 53769 | 10522 | | 5.96 | | 0.015 |
| **Retailer Type** |  |  | |  | |  |
| Map-based | Ref |  | |  | |  |
| Browser-based | 50290 | 11442 | | 16.76 | | <0.001 |

**Supplemental Table 4:** Association between flavored and inhalable derived cannabinoid product availability and average monthly website traffic, adjusting for retailer type, among online vape shops serving San Diego, California, from March to August 2023, that sell derived cannabinoid products (n=39).

|  | Beta | Standard error | | t value | | p-value |
| --- | --- | --- | --- | --- | --- | --- |
| **Inhalable and Flavored Cannabinoid Products Offered** |  |  |  | |  | |
| No | Ref |  | |  | |  |
| Yes | -94779 | 30116 | | 1.85 | | 0.17 |
| **Retailer Type** |  |  | |  | |  |
| Map-based | Ref |  | |  | |  |
| Browser-based | 119054 | 32165 | | 32.09 | | <0.001 |
